# Supplementary material for: Hybrid Models and Biological Model Reduction with PyDSTool
Source: PLoS Comput Biol. 2012 Aug 9;8(8):e1002628. doi: 10.1371/journal.pcbi.1002628 (PMC3415397; doi:10.1371/journal.pcbi.1002628)
Supplement: Text S4 — Complete source code for the PyDSTool package (version 0.88.120504). Includes API documentation and help files linking to web pages. This file is identical to the current public release on Sourceforge.net. (ZIP) [file pcbi.1002628.s004.zip › PyDSTool/html/PyDSTool.Toolbox.phaseplane.distance_to_pointset-class.html]

xml version="1.0" encoding="ascii"?


PyDSTool.Toolbox.phaseplane.distance\_to\_pointset


| Home | Trees | Indices | Help | PyDSTool |
| --- | --- | --- | --- | --- |

Package PyDSTool ::
Package Toolbox ::
Module phaseplane ::
Class distance\_to\_pointset

[hide private]

[frames] | no frames]

# Class distance\_to\_pointset

source code

```
object --+
         |
        distance_to_pointset
```

---

```
First and second maximum and/or minimum distances of a point q
to a set of points, returning a dictionary keyed by 'min' and
'max' to dictionaries keyed by integers 1 and 2 (respectively).
The values of this dictionary are dictionaries of
  'd' -> distance
  'pos' -> index into pts

To restrict the search to a lower-dimensional subspace, specify a sub-set
of the variables in q. Defaults to Euclidean distance (normord=2),otherwise
specify metric-defining norm order.

To speed up the search, use n > 1 to create n segments of the pointset,
from which representative points will first be assessed, before the search
narrows to a particular segment. Be sure to use a large enough n given
the variation in each segment (which will otherwise cause spurious results
for certain test points). Default n=30.
[NB This option currently only returns the first max/min distances]

The radius option provides an opportunity to use information from
a previous call. This works for minimum distances only, and forces
the same segment to be searched, provided that the new point is
within the radius of the previous point (using 2-norm unless a 2D
Point is specified). CURRENTLY, THIS DOES NOT WORK WELL. KEEP IT
SWITCHED OFF USING (default) radius=0.

If gen is not None, it should contain a generator for use with isochron-related
events, for more accurate computations. The remaining arguments are associated
with this usage.
```


|  |  |
| --- | --- |
| Instance Methods | [hide private] |

|  |  |
| --- | --- |
| \_\_init\_\_(self, pts, n=30, radius=0, gen=None, iso\_ev=None, other\_evnames=None, pars\_to\_vars=None)  x.\_\_init\_\_(...) initializes x; see x.\_\_class\_\_.\_\_doc\_\_ for signature | source code |

|  |  |
| --- | --- |
| clear\_history(self) | source code |

|  |  |
| --- | --- |
| \_\_call\_\_(self, q, use\_norm=False, normord=2, minmax=`[``'``min``'``,` `'``max``'``]`) | source code |

|  |  |
| --- | --- |
| \_distance(self, q, use\_norm, normord, minmax) | source code |

|  |  |
| --- | --- |
| search\_both(self, q, pts, fn, le, ge, dmin, dmin\_old, dmax, dmax\_old) | source code |

|  |  |
| --- | --- |
| search\_min(self, q, pts, fn, le, dmin, dmin\_old) | source code |

|  |  |
| --- | --- |
| search\_max(self, q, pts, fn, ge, dmax, dmax\_old) | source code |

**Inherited from `object`**:
`__delattr__`,
`__getattribute__`,
`__hash__`,
`__new__`,
`__reduce__`,
`__reduce_ex__`,
`__repr__`,
`__setattr__`,
`__str__`


|  |  |
| --- | --- |
| Class Variables | [hide private] |

\_keys = `['d', 'pos']`


|  |  |
| --- | --- |
| Properties | [hide private] |

**Inherited from `object`**:
`__class__`


|  |  |
| --- | --- |
| Method Details | [hide private] |

|  |  |
| --- | --- |
| \_\_init\_\_(self, pts, n=30, radius=0, gen=None, iso\_ev=None, other\_evnames=None, pars\_to\_vars=None)  *(Constructor)* | source code |

x.\_\_init\_\_(...) initializes x; see x.\_\_class\_\_.\_\_doc\_\_ for
signature

Overrides: object.\_\_init\_\_: *(inherited documentation)*

  


| Home | Trees | Indices | Help | PyDSTool |
| --- | --- | --- | --- | --- |

|  |  |
| --- | --- |
| Generated by Epydoc 3.0.1 on Fri May 4 15:24:09 2012 | http://epydoc.sourceforge.net |
